# Not all minds that wander are lost: the importance of a balanced perspective on the mind-wandering state

**DOI:** 10.3389/fpsyg.2013.00441

**Published:** 2013-08-16

**Authors:** Jonathan Smallwood, Jessica Andrews-Hanna

**Affiliations:** ^1^The Department of Social Neuroscience, The Max Planck Institute of Human Cognitive Brain SciencesLeipzig, Germany; ^2^The Institute of Cognitive Science, University of Colorado BoulderBoulder, CO, USA

**Keywords:** mind-wandering, S, daydreaming, stimulus independent thought, task unrelated thought, self-generated thought

## Abstract

The waking mind is often occupied with mental contents that are minimally constrained by events in the here and now. These self-generated thoughts—e.g., mind-wandering or daydreaming—interfere with external task performance and can be a marker for unhappiness and even psychiatric problems. They also occupy our thoughts for upwards of half of the time, and under non-demanding conditions they (i) allow us to connect our past and future selves together, (ii) help us make successful long-term plans and (iii) can provide a source of creative inspiration. The lengths that the mind goes to self-generate thought, coupled with its apparent functionality, suggest that the mind places a higher priority on such cognition than on many other mental acts. Although mind-wandering may be unpleasant for the individual who experiences it and disruptive to the tasks of the moment, self-generated thought allows consciousness freedom from the here and now and so reflects a key evolutionary adaptation for the mind. Here we synthesize recent literature from cognitive and clinical psychology and propose two formal hypotheses that (1) highlight *task context* and *thought content* as critical factors that constrain the costs and benefits of self-generated thought and (2) provide direction on ways to investigate the costs and benefits from an impartial perspective.

When reading this article there may be moments when the prose that makes up the narrative does not form the basis of you thoughts. Some of these thoughts may seem trivial: Did you lock your car? Perhaps you should check your phone to see if you received an email? Others may entail ideas with implications for your life—who you will marry, what course you will take, or why you want to pursue a career in science. First studied almost fifty years ago (Singer and Antrobus, 1963, 1965; Klinger, 1966, 1967; Klinger and McNelly, 1969), in the last decade these self-generated thoughts (aka mind-wandering or daydreaming) have become an important topic of research (Raichle et al., 2001; Smallwood and Schooler, 2006; Mason et al., 2007).

At times we can all find our minds' tendency to self-generate thought unrelated to what we are doing annoying, and this intuition is supported by a growing body of research. Self-generated thought occupies our thoughts when our mood is low (Smallwood et al., 2009a) and can contribute to future unhappiness (Killingsworth and Gilbert, 2010). Task unrelated self-generated thought is also disruptive to the goals of the moment: It interferes with the models we build when we read a novel (Smallwood et al., 2008; McVay and Kane, 2012a,b), reduces external vigilance (McVay and Kane, 2009, 2012a,b) and its occurrence can influence estimates of a person's intelligence (Mrazek et al., 2012). Together these qualities provide us with a strong impression that self-generated thought is an unintended lapse because we all want to be happy and to perform tasks to the best of our abilities.

Despite our sense that self-generated thought can be an irritant, this interpretation is unnecessarily simplistic. Humans spent almost half of the day engaged in the experience (Klinger and Cox, 1987–1988; Killingsworth and Gilbert, 2010) and any behavior that occupies such a large amount of time is a strong candidate for consideration as normal, if not important. Indeed, over the last two decades neuroimaging has revealed that almost all of the neural systems that are employed in the service of explicit external tasks exhibit spontaneous activity during the resting state (Biswal et al., 1995; Smith et al., 2009). Thus while we may find mind-wandering frustrating, both neural and experiential evidence demonstrates that mental activity that is independent from external stimulation is a normal rather than an exceptional state.

The situation is even more complex once it is recognized that self-generated thought has demonstrable benefits. Mind-wandering often involves a focus on our future or reflections about our past (Smallwood et al., 2009b; Smallwood and O'Connor, 2011; Stawarczyk et al., 2011), and such forms of mental time-travel allow us to devote conscious thought to the connections between our past and future selves and who we are now (Tulving, 1987; Suddendorf and Corballis, 2007). Self-generated thought can enhance creativity (Baird et al., 2012), helps consolidate self-memories (Smallwood et al., 2011) and is linked to a style of long-term decision making characterized by patience rather than impulsivity (Smallwood et al., 2013). Mind-wandering is thus associated with skills like creativity, planning and delaying gratification and so reflects capacities that are necessary to navigate the complex social environment in which we exist (Frith and Frith, 2007).

Evidence that self-generated thought is normal, coupled with its apparent functionality suggests that our natural inclination to dismiss mind-wandering as a lapse is more simplistic than it is realistic. This paper develops the idea that the current view of mind-wandering employed by many researchers is unnecessarily narrow and considers this argument with specific reference to the methodology that studies employ, the focus of intervention studies and the conceptual perspectives with which the phenomenon that self-generated thought is approached. By developing these themes we propose two formal hypotheses that (1) highlight *task context* and *thought content* as critical factors constraining the costs and benefits of self-generated thought and (2) provide direction on ways to investigate the costs and benefits from an impartial perspective.

## The methodological perspective: context matters

When investigating any particular psychological phenomena the experimenter must choose a context in which to embed his or her research, and this decision is important because it can influence the results and impact upon the interpretations of the data. There are many factors that affect the choice of a particular paradigm, and there is one that is especially important when choosing how to conduct a mind-wandering experiment: *the extent to which the task paradigm in which self-generated thought is measured demands continuous attention*. There is a recent trend in research on mind-wandering to use experimental contexts that assess task unrelated thinking in the context of complex tasks (such as encoding, reading, an n-back working memory task, or measures of fluid intelligence: Smallwood et al., 2003, 2008; McVay and Kane, 2009, 2012a,b; Mrazek et al., 2012). These tasks demand attention in a continuous manner and are in sharp contrast to earlier research into mind-wandering which favored simpler tasks (Antrobus et al., 1967). Although in both cases experimenters are interested in exploring the experience of task unrelated thought, these different experimental conditions offer quite different advantages vis-a-vis their utility as a context to measure our capacity to generate thought that does not arise from sensory input.

Complex demanding tasks provide the opportunity to assess mind-wandering in a context in which the experience has outcompeted a pertinent external task, thus allowing the experimenter the chance to gauge the deleterious consequences on task performance. They are also a context when the mind-wandering experience is relatively uncommon and in which a conscientious and motivated participant is likely to be inclined *against* engaging in self-generated thought. By contrast, simple undemanding tasks require few cognitive resources and allow for a more frequent mind-wandering experience. Simple tasks are also closer to the environment in which we are likely to experience most self-generated thoughts in daily life (e.g., when we are stuck in a traffic jam, jogging, or are brushing our teeth).

The choice of a difficult or an easy task paradigm leads to non-trivial differences in experimental results and thus influences the interpretations that the authors place on the mind-wandering state. For example, in complex demanding experimental conditions, task unrelated thoughts lead to significant disruptions in behavioral performance, including absent minded forgetting (Smallwood et al., 2003), poor reading comprehension (Smallwood et al., 2008; Reichle et al., 2010), and poor encoding of material into long-term memory (Smallwood et al., 2003). By contrast, almost without exception, all of the adaptive features of self-generated thought are observed in less demanding situations: greater freedom to consider the future (Smallwood et al., 2009b), greater opportunity to think creatively (Baird et al., 2012), and an association with economic choices that are patient rather than impulsive (Smallwood et al., 2013). Importantly in both contexts the dependent measure is the same: the assessment of task unrelated thoughts based on participants' self-reports. Whereas authors conducting experiments in complex task environments are justified in describing task unrelated thought as a lapse of attention, experimenters using simpler task environments are justified in describing their results as evidence of the capacity of self-generated thought to facilitate many of the features that are critical to the human condition. A simple difference in the task context, thus, leads to radical differences in how the experience is interpreted by the researcher and thus the perspective from which scientists, and society at large, view the mind-wandering state.

These contextual differences in the correlates of task unrelated thought occur because the experience is an example of a psychological state. Just as different behavioral states cannot be related to different personality traits without taking into context the situation in which behavior evolves (Mischel and Ayduk, 2002), the self-generated thoughts and feelings that people experience are not independent of the context in which they occur. Mind-wandering under conditions when the external environment demands attention carries with it significant risk and so when task unrelated thought occurs under these conditions it is more likely to signify performance disruptions, cognitive problems, risk taking or low motivation to perform a task. By contrast, when the environment lacks compelling inputs, the risks associated with self-generated thought are substantially reduced and not devoting cognitive resources to the environment allows the motivated individual the chance to consider longer term goals and objectives. The lack of independence of key features of the mind-wandering experience from the context in which it is measured indicates that certain first order claims regarding the relationship between self-generated thought and other variables will not generalize to every situation.

These findings lead to our first hypothesis surrounding the factors that constrain the costs and benefits of self-generated thought. According to the context-regulation hypothesis (Box 1), *the capacity to regulate the occurrence of self-generated thought so as to minimize the risk of the experience derailing on-going task performance is indicative of a cognitive system that is functioning in an adaptive manner*. Here we are interested in the concept of adaptive behavior in the sense of a well-adjusted cognitive system rather than in a strictly evolutionary sense. In addition to contextual influences on ongoing behavioral performance, the context-regulation hypothesis makes an important prediction about individual variability in the adaptive nature of self-generated thought. In particular, it predicts that individuals who are able to *limit its occurrence to non-demanding situations* will experience the fewest costs and the greatest benefits of self-generated thought. Indeed, existing studies provide initial support for this prediction: individuals who mind-wander to a greater degree under non-demanding conditions and/or to a lesser degree under demanding conditions have a higher working memory capacity (Kane et al., 2007; McVay and Kane, 2009; Levinson et al., 2012) greater creativity (Baird et al., 2012), and demonstrate patient and prospective as opposed to impulsive and immediate choices (Smallwood et al., 2013).

Box 1 **The Context Regulation Hypothesis.**

The capacity to regulate the occurrence of self-generated thought (SGT) so as to minimize the risk of the experience derailing on-going task performance is indicative of a cognitive system that is functioning in an adaptive manner.

- **SGT is most common in non-demanding contexts**.
  Self-generated thought occurs less frequently when individuals perform demanding external tasks (e.g., Antrobus et al., 1967, 1970; Teasdale et al., 1995; Smallwood et al., 2004, 2007, 2009b; Mason et al., 2007).
- **The costs and benefits of SGT are context dependent**.
  SGT under conditions that demand continuous attention is unproductive because it can be a source of error (McVay and Kane, 2009, 2012a,b; Mrazek et al., 2012).
  SGT under non-demanding situations is beneficial because it is associated with capacities such as creativity, patience and better cognitive control (Baird et al., 2011, 2012; Levinson et al., 2012; Smallwood et al., 2013).
- **Experimental Considerations**.
  Since the complexity of a given task environment is always relative, self-generated thought should be measured across a range of different contexts.
  The relative cognitive capacities of the individual will determine the extent to which he/she is able to successfully perform more complex tasks while simultaneously engaging in self-generated thought.

Rather than assuming that the task context does not matter, as research has often tended to do, future studies should attempt to measure the experience across different conditions and to provide detail regarding how mind-wandering relates to different psychological features in different contexts. Ideally these task environments should be varied systematically in terms of cognitive processes that are thought to influence the mind-wandering state. Examples might include varying the complexity of perceptual input (e.g., Forster and Lavie, 2009) the extent of practice with the task (Teasdale et al., 1995; Mason et al., 2007), the amount of working memory allocated to the task (Smallwood et al., 2009b). By looking simultaneously across carefully chosen task conditions, researchers are not limited to making general statements regarding how certain types of psychological states relate to the mind-wandering experience. Instead, by assuming that the context matters, researchers can design experiments to allow them to make targeted statements regarding the conditions under which individuals' pre-dispositions toward particular types of mind-wandering under specified task conditions relate to particular psychological costs or benefits.

## The clinical perspective: content matters

The complex nature of the mind-wandering phenomena demands that we adopt a more nuanced view of self-generated thought and this is especially important for relating the concept to psychological wellbeing. In addition to the context with which self-generated thought occurs, an additional element of mind-wandering that is likely to be important to consider is the content underlying the experience (Watkins, 2008). Profiles of thought content often vary widely across individuals, and this variability must be taken into consideration before making general conclusions about the adaptive nature of self-generated thought. While some forms of thought content are linked to maladaptive outcomes including psychological distress and unhappiness, other forms highlight the adaptive nature of the experience.

Evidence supporting an unconstructive view of self-generated thought comes from studies linking the mind-wandering state to unhappiness (Killingsworth and Gilbert, 2010) and mind-wandering about the past to negative mood (Smallwood and O'Connor, 2011). Of clinical relevance, repetitive thoughts focused on negative, self-related content represent core features of depressive rumination—a style of thinking elevated in individuals with depression and anxiety (Nolen-Hoeksema, 2000; Watkins, 2008). Depressed and suicidal individuals also exhibit over general memories and future thoughts with fewer episodic details (Raes et al., 2005; Williams et al., 2007). Collectively, these findings foster the sentiment that self-generated thoughts are maladaptive because they impede happiness and reduce psychological well-being.

However, these findings stand in stark contrast to a growing number of studies revealing that in non-clinical populations, self-generated thought is characterized by content of a far more adaptive and constructive nature (reviewed in Smallwood and Schooler, 2006; Baars, 2010; Andrews-Hanna, 2012; Smallwood, 2013a,b). The finding that self-generated thought is highly self-relevant is consistent with the notion that such thoughts provide a means to focus on and solve one's current concerns (Klinger, 1971, 2009; Smallwood et al., 2011). On a related note, self-generated thoughts also facilitate insight and creative problem solving (Baird et al., 2012), and in contrast to depressed patients they are often positive in valence (Killingsworth and Gilbert, 2010) and marked by a moderate amount of visual imagery (Delamillieure et al., 2010). Importantly, the temporal dynamics of self-generated thought also speaks to its adaptive potential. A majority of individuals' thoughts are spent engaged in mental time-travel, particularly oriented toward the future and in pursuit of future goals (Smallwood et al., 2009a,b; Smallwood and O'Connor, 2011; Andrews-Hanna et al., 2010; Baird et al., 2011; Stawarczyk et al., 2011; Song and Wang, 2012). From an evolutionary perspective, prospection allows us to simulate plausible outcomes to alternative future events, including the emotional states of ourselves and other people in response to such events (Gilbert and Wilson, 2007). In this way self-generated thought is an adaptive process that helps us select the optimal course of action, prepare for upcoming events, and achieve our upcoming goals (Schacter et al., 2007; Suddendorf and Corballis, 2007; Suddendorf et al., 2009; Szpunar, 2010). More broadly, mental time travel about the past and future provides us with a sense of self-identity and continuity across time (Tulving, 1985; Prebble et al., 2013). Additionally, both prospective and past-oriented thoughts are often characterized by a recent and immediate focus (Spreng and Levine, 2006; Andrews-Hanna et al., 2010), inviting the speculation that such thoughts provide a means to consolidate recent and upcoming experiences into long-term memory (Wamsley and Stickgold, 2010; Andrews-Hanna, 2012).

The relationship between the content of self-generated thought and mood may be even more complex. Recent work exploring the temporal dynamics of the link between the content of self-generated thought, and mood demonstrate that the relationship may have a complex temporal relationship. Using time lag analysis, Ruby et al. (under revision) demonstrated that the occurrence of past related off task thought while participants performed a simple non-demanding task was associated with subsequent reports of negative mood. By contrast, self-generated thought focused on the future was associated with a subsequent more positive mood. Similarly, Franklin et al. (in submission) demonstrated that in daily life when self-generated thoughts are rated as more interesting, mood was more positive. These findings suggest that the content of self-generated thought is important in determining its consequence on subsequent mood.

Based on these findings, we propose our second hypothesis regarding factors that constrain the costs and benefits of self-generated thought. The content regulation hypotheses (Box 2) suggests that *the relationship between self-generated thought and psychological wellbeing depends on assessing how individuals regulate the content of their mental experiences so as to maximize thoughts with a productive outcomes, and minimize those which are detrimental to their happiness or other life outcomes.* This hypothesis assumes that the mind-wandering is a heterogeneous, rather than a homogeneous state, and thus it might not make sense for intervention studies to seek to reduce self-generated thought per se. Instead techniques could focus on changing the *content* of the experience that is disruptive to an individual's health and well-being without impacting upon the obvious benefits that self-generated thought conveys to the individual. For example, interventions aimed at reducing negatively-valenced, retrospectively focused, or abstract repetitive styles of thinking using mindfulness-based or other cognitive therapeutic approaches may help break the cycle linking off task thought to transient and long-lasting unhappiness (Teasdale et al., 2002). It would also be of interest to identify whether training that favors mindfulness alters the content of mind-wandering as well as its occurrence. A clear research priority, however, should be the non-judgmental gathering of normative data on how different features of mind-wandering levels vary across situations, cultures and psychological conditions to provide empirical information on what constitutes abnormal content for self-generated thought and what forms of content help the individual fulfill his or her personal goals.

Box 2 **The Content Regulation Hypothesis.**

The capacity to regulate the content of self-generated thought (SGT) so as to maximize the productivity of the experience is indicative of a cognitive system that is functioning in an adaptive manner.

- **SGT focused on the future may allow individuals to anticipate and plan their futures**.
  In normative terms SGT is focused on the future and oriented toward one's personal goals (Smallwood et al., 2009b; Andrews-Hanna et al., 2010; Baird et al., 2011; Song and Wang, 2012).
- **SGT focused on the past can be a indicative of distress and unhappiness**.
  SGTs are often oriented toward the past and focused on negative topics under conditions of unhappiness (Smallwood and O'Connor, 2011; Stawarczyk et al., 2013).
- **Experimental considerations**.
  The adaptive value of different thought content may vary across situations. Ruminating on the past thought maybe generally unproductive, whereas if future related thought takes the form of worry it could also be problematic.
  The adaptive value of SGT can also depend on the personality and cognitive capacities of the individual.

### Moving forward: towards a nuanced understanding of the mind-wandering state

Complex problems have simple easy to understand wrong answers.

*Henry Louis Mencken (1880–1956)*

Empirical work conducted over the last decade has realized the psychological costs and benefits associated with mind-wandering and it is important that this research continues. In order to move beyond this descriptive analysis of the experience of mind-wandering, however, it is necessary to embrace the fact that phenomena as intricate as self-generated thought require explanations that are sufficiently complex. Recognizing that self-generated thought depends on multiple cognitive processes and is thus dependent on an individual's profile of affective traits, cognitive ability, or motivation requires that we should acknowledge that not every experimental finding on mind-wandering should generalize across every person and situation. With this knowledge in hand, we should design our experiments so that we can elucidate the scientific principles that explain how task contexts interact with individual differences to produce different forms of self-generated thought. Likewise evidence that mind-wandering is associated both with costs *and* benefits require that we design intervention studies carefully so that we curtail only those aspects of mind-wandering that impair psychological wellbeing. Finally, at a conceptual level we need to acknowledge that the capacity to generate thoughts that do not arise from the environmental input to which we are exposed is far more than a failure to constrain attention to perception; it reflects an evolutionary adaptation that allows agents to perform actions that are not simply a reflexive response to the outside world. The capacity for freedom from immediacy prevents our thoughts from being tied to events in the perceptual moment and self-generated thoughts that are not related to environmental input are simply highly developed example of this highly valuable cognitive capacity. As *experiencers* we may understand mind-wandering as an unintended lapse; as *scientists* we should take the study of self-generated thought seriously because it can reveal core features of how thinking operates.

### Conflict of interest statement

The authors declare that the research was conducted in the absence of any commercial or financial relationships that could be construed as a potential conflict of interest.
